# Research ethics: a safeguard for advanced technologies

**DOI:** 10.1093/nsr/nwz133

**Published:** 2020-10-16

**Authors:** Hepeng Jia

**Affiliations:** Professor of science communication, Soochow University and a freelancing science writer for NSR

## Abstract

With the fast development of cutting-edge technologies and their greater integration into human life, more ethical challenges emerge. The problem became more salient when the world's first genetically edited babies were born in China in violation of existing ethical rules. Although the responsible researcher He Jiankui was sentenced for imprisonment for three years last December, it is still necessary to examine the current status of research ethics and the challenges in China. Has China set up a sophisticated research ethics system? For research ethics and their implementation in China, are there unique national characteristics? Can the dominant ethics principles primarily developed from life science research be equally adopted in the emerging artificial intelligence research and development? At an online forum organized by National Science Review (NSR) and through subsequent correspondences among forum participants, NSR Executive Editor-in-Chief Mu-ming Poo and guest moderator Hepeng Jia asked three scientists and three bioethicists or philosophers of science and technology in the field to examine the dynamic development of research ethics in China.

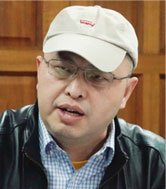

Weiwen Duan

Philosopher of Science and Technology at Chinese Academy of Social Sciences, Beijing, China

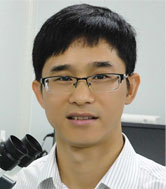

Junjiu Huang

Life scientist focused on genetics at Sun Yat-sen University, Guangzhou, China

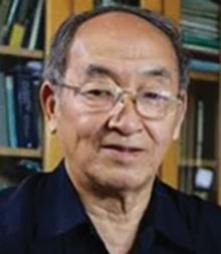

Renzong Qiu

Bioethicist at Chinese Academy of Social Sciences, Beijing, China

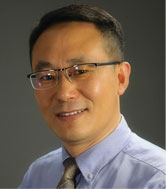

Qiang Sun

Life scientist and the principal investigator (PI) of clone monkey program at Shanghai Institute of Neuroscience, Chinese Academy of Sciences, Shanghai, China

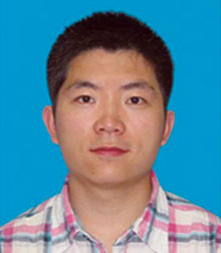

Yi Zeng

Artificial intelligence scientist at Institute of Automation, Chinese Academy of Sciences, Beijing, China

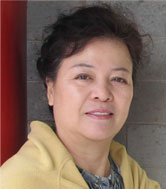

Xiaomei Zhai

Bioethicist at Chinese Academy of Medical Sciences/Peking Union Medical College, Beijing, China

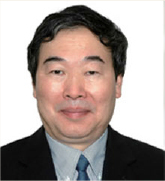

Mu-ming Poo (Chair)

Neurobiologist at Center for Excellence in Brain Science and Intelligence Technology, Chinese Academy of Sciences, Shanghai, China

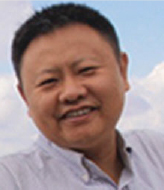

Hepeng Jia (Co-chair)

Professor of Science Communication at Soochow University, Suzhou, China

## CURRENT SITUATION OF RESEARCH ETHICS IN CHINA

**Poo:** In recent years, research ethics have become an important and hot issue. Since China's science and technology (S&T) have achieved significant progress in areas of genome editing and artificial intelligence (AI), we also face significant ethical challenges [1]. Now let us examine the basic situation and challenges in these fields.

**Zeng:** AI is a disruptive technology [2]. We must ensure that disruptive technologies like AI are developing in a direction that is beneficial to society. In the process of AI development and applications, we need to do our best not to bring high risks and introduce unpredictable problems into the community.

It is necessary to develop specific regulations for each emerging technology. The appropriate approach to developing emerging technologies should insist on ‘ethical thinking ahead’.

—Renzong Qiu

**Huang:** Let me talk about genetic editing. I think the most important thing is that whether in genetic or AI or brain science, the speed of development in S&T may far exceed that of ethics. Some technological developments may have a very violent collision with ethics.

Companies are developing treatments for genetic or other diseases. But meanwhile, ethics, including our medical procedures, and some rules and regulations such as patient's consensus, have not been mature, so there are more problems.

**Poo:** Genome editing as an emerging technology has involved the safety issue. Meanwhile, ethically speaking, whether the human embryo genome can be edited is another issue. They are different but mutually connected. Let us further discuss them.

**Qiu:** We have been discussing genetic ethics for decades and have formed several regulations and norms, but still, there is a regulatory gap [3]. The developing rules have not caught up with the rapid development of cutting-edge technologies. It is necessary to develop specific regulations for each emerging technology. The appropriate approach to developing emerging technologies should insist on ‘ethical thinking ahead’.

**Duan:** I fully agree with ‘ethical thinking ahead’. The development of technology has become more and more refined, and then it has different problems in different fields, and many issues are special. Therefore, I think that in the aspect of the integration of technology and ethics, it is essential that we combine some specific ethical problems with scientific research.

For example, before AI’s widespread application, we must be assured as to whether such an algorithm is interpretable and whether it has transparency, and so on. These are because regulating AI cannot just stay on the abstract ethical norms.

**Poo:** How about the situation in AI in China? Professor Zeng, can you introduce its condition to us?

**Zeng:** I think that the ethical issues of AI may have different perspectives than life science ethics. I believe life science research has ethical guidelines we should follow when doing experiments. AI ethics is not only about the researcher who is engaged in AI research but is essential in the whole life cycle of the research, development and application of AI.

AI research and applications affect society from different angles and at various degrees [4]. Therefore, we must regulate not only AI researchers but also their products. Our AI models, applications and algorithms should comply with our human society's moral and ethical norms.

AI development needs a strategic vision of where we are going. I think this has been extensively discussed in China. The Ministry of Science and Technology of China has set up a National Governance Committee for the New Generation AI. On 17 June 2019, the committee released the Governance Principles for the New Generation Artificial Intelligence (GPNGAI). GPNGAI emphasizes using AI as an enabling technology to support the UN sustainable development goals and promotes harmony as a core principle.

**Poo:** These are excellent discussions. Now let us turn to Professor Sun on the ethical issues about animal use.

**Sun:** Regarding the ethics of experimental animals, my personal views are that regardless of cultural backgrounds, we should base ethical disciplining on science. Many experiments we do with experimental animals and tests on animals are first to solve human problems [5,6].

At our institute, we have followed the general accepted rules of animal protection, such as 3R principles—Replacement, Reduction and Refinement. Nationwide, the policy framework to protect experimental animals has been set up, and most research institutions have set up their animal welfare committees. However, some institutions may not have strictly implemented the ethical reviews regarding animal use.

## SETTING UP A ROBUST POLICY FRAMEWORK FOR RESEARCH ETHICS

**Poo:** We are particularly concerned with the future of gene editing. How to deal with ethical issues as the technology advances? In terms of management, there should be further improvement. How can we get a consensus? What kind of countermeasures could be introduced in China against the unethical practice?

**Duan:** After the scandal of He Jiankui, science institutions ranging from the ethics committee of the Chinese Academy of Sciences to the China Association for Science and Technology (CAST) have appealed to set up a top expert group on research ethics. Currently, the Chinese leadership has approved to set up a national committee on research ethics. Besides, a recent seminar on the revision of S&T progress law suggested that the amendment of the fundamental law governing S&T activities in China will add new articles on research ethics. This amendment will provide a legal foundation for research ethics governance.

**Poo:** But if the rules of ethics could not be rigorously implemented and supervised, without strict punitive actions to violators, we are likely to find more cases of ethical breaches.

**Qiu:** So why do some people not abide by the laws? The Ministry of Health (MOH, now the National Health and Family Planning Commission) had detailed rules on ethical review of biomedical research involving human subjects, but these rules were not effectively enforced. Some avoided it because of their conflict of interests, and they wanted to prevent these rules for their benefit. Right? So for developing new or emerging technologies, no matter what they are, you have first to make the rules based

AI research and applications affect society from different angles and at various degrees. Therefore, we must regulate not only AI researchers but also their products.

—Yi Zeng

If the rules of ethics could not be rigorously implemented and supervised, without strict punitive actions to violators, we are likely to find more cases of ethical breaches.

—Mu-ming Poo

on ethical inquiry. So we call this approach ‘ethically thinking ahead’.

**Zhai:** We have repeatedly stressed that China has regulations. They include the assisted reproduction rule in 2003, the 2004 human stem cell regulation issued jointly by the Ministry of Science and Technology (MOST) and the MOH, and other regulations. The 2003 assisted reproduction rule prohibited research on the human embryo for productive purposes during the 14-day limit. Why do we still have so many violations? The lack of ethical review and governance capacity is one major reason.

Therefore, the government now has a clear stance on the issue: to govern it hierarchically. In the past, we handed over the power of ethics review and approval of a protocol only to the institutional ethics review committee, but the capability of such a committee was uneven among various institutions. So now, China will adopt the approach of risk-based regulation. Research involving medium and high-level risks must be subject to a higher level of review and approval, i.e. the review and approval of the provincial/municipal ethics committee or even ministerial ethics committee.

This hierarchical governance will soon be implemented. Another factor leading to violations is the bureaucratic fragmentation. Some rules are under the jurisdictions of MOST, Ministry of Education or MOH. In this case, we should make a seamless connection between these ministries. Otherwise, there are some loopholes. I think these measures will make up for these vulnerabilities.

**Qiu:** Sure. Military hospitals and institutions should be better supervised too.

**Zhai:** That is correct. Universal state policy should widely supervise research activities sponsored by MOST, MOH, Ministry of Education and military systems.

**Qiu:** I want to supplement one thing. It is not just about how the governmental regulators oversee, but also about more general research and social policies. The policies that only encourage scientists to make money should be revoked. Policymakers should understand that such policies trap scientists in interest conflicts.

## OVERCOMING IMPLEMENTATION LOOPHOLES

**Poo:** How can we make sure the new system for research ethics governance is effectively implemented?

**Qiu:** I want to supplement this implementation question from another viewpoint. Why have we not paid enough attention to the formulation of relevant ethical norms or regulations in the past? There are some perceptual obstacles.

First, some policymakers think that it is time to advance science, rather than discussing ethical issues. When I was invited by scientists to participate in drafting the 863 (state high-tech development) Program, some policymakers held this view. Now, does this ‘not the right time’ thinking still influence policymakers? Maybe.

Second, some policymakers always feel that ethics is opposed to science as if ethical regulations hinder our S&T development. Sometimes it is said that foreign oversight is strict, so a looser regulatory system can help create opportunities for China to speed up the S&T development.

**Duan:** Yes. Some Chinese scientists and policymakers still think that looser ethical requirement is a competitive advantage for Chinese science. Besides, many scientists consider the safety issues of emerging technologies have been solved. The public and ethicists’ worries can harm scientific development.

On the other hand, policymakers would not want to see research ethics issues evolve into scandals of China's S&T management. So, the heated public discussion on some scandals may not have been encouraged. Meanwhile, some individual research institutions and scientists are often apathetic to bioethics rules. In a sense, this behavior has reflected an unhealthy scientific culture in China.

**Sun:** Many Chinese science funding agencies such as MOST have adopted rules that require an ethical review of experimental animal use in grant applications, but these reviews are easy to pass. Besides, some ethical review committees do not have the qualification to perform the review. Therefore, if we want to assure the proper observance of the ethical regulations, there must be a rigorous regulatory examination. The regulators should also have follow-up monitoring for the implementation.

Other regulatory measures are essential too. You cannot just rely on self-appointed ethical review committees. The certification of these committees is critical. China has begun to adopt a CNAS (China National Accreditation Service for Conformity Assessment) certification system. The accreditation service of the authoritative AAALAC (Association for Assessment and Accreditation of Laboratory Animal Care) is also available in China. In addition to certifications, supervision of experimental animal uses and accreditation of their lab management by third parties are essential.

**Jia:** Regarding implementation, we noticed that He Jiankui's rogue practice received the approval of the institutional review board (IRB) of the Shenzhen hospital where the two babies were

Therefore, the government now has a clear stance on the issue: to govern it hierarchically. This hierarchical governance will soon be implemented.

—Xiaomei Zhai

There should be a higher level of committee in charge of the ethical evaluation of this emerging research. The soon-to-be-launched National Science and Technology Ethics Committee should take this role.

—Weiwen Duan

born. But apparently, the IRB members of this local hospital did not have necessary knowledge regarding genome editing.

**Duan:** It is not appropriate for the local hospital to perform the ethical review of the cutting-edge research. They do not have the necessary expertise and knowledge to fulfill the task. The IRBs of average research institutions are more suitable to do ethical reviews for regular research. There should be a higher level of committee in charge of the ethical evaluation of this emerging research. The soon-to-be-launched National Science and Technology Ethics Committee should take this role. The top leadership has approved its establishment, and its composition has been nearly completed.

**Jia:** Professor Zhai mentioned the universal and hierarchical governance of research ethics in our discussion above, which should include establishing such a committee. What are the implications of setting up such a national committee in assuring the better implementation of ethical reviews in practice?

**Duan**: The exact function and jurisdiction of such a committee are still under discussion, but one thing is clear. It will encourage concrete research institutions and local S&T authorities to pay more attention to research ethics. Their ethical review body will be more formal and cover more comprehensive issues. Such a national committee cannot fill all the possible loopholes in the ethical regulation of research. There might still be some researchers who violate established rules. Nevertheless, in terms of providing behavior norms in emerging research areas and punishing wrongdoers, the committee is expected to play a more prominent role. It can also have prior consideration of possible ethical violations and help set up a warning system.

## THE VOICE OF CHINA IN THE GLOBAL GOVERNANCE OF RESEARCH ETHICS

**Poo:** There are the same ethical standards for all human beings all over the world. On the other hand, ethical issues may have distinct flavors depending on cultural and religious differences. For example, people with diverse religious backgrounds have different views about induced abortion. The legislation on induced abortion in various states of the US is different. So, how do we meet such cultural differences in formulating ethics rules that have universal consensus?

**Qiu:** One of the Human Genome Project's findings is that all human races and ethnic groups are similar under the skin. It constitutes robust scientific evidence to prove Confucius’ saying, ‘Human nature is similar; only the practices make people far away’. People in different countries used to be isolated from each other, but we are now so close to each other, and we have a common understanding. We have the Nuremberg Code, which was initially drafted against the Nazi's inhumane, unethical and anti-humanity experiments on human beings. We all agree with the basic principles of the code.

In the Nuremberg Code, there are two fundamental principles to protect human subjects in medical research. One is that your research must be beneficial to society and useful to human beings. We should minimize potential risks and maximize possible benefits. Second, if you want candidates of human subjects to participate in research, you must get the consent of the person. You have to tell him or her all the information about the investigation, help them comprehend the data, and then they should freely make their own decision, and voluntarily participate in the study.

Based on these principles, IRB gradually evolved, and an external review was performed by other scientists, ethicists, jurists and other experts as well as a representative of laypeople. It would be ridiculous if one stresses China's cultural characteristics to reject these international consensuses and norms.

**Huang:** China has already achieved significant progress in science. It has the right of speech in many scientific issues, but its power to express in ethical matters is something we should consider. If China has her voices heard, perhaps it is easier to make something new in science.

The state must have a system to encourage everyone to discuss ethical issues, and then develop a standard acceptable to the public. Then with this standard, we may be able to create a better environment for emerging technology development. If we return to the area of human reproduction, one will see that China's voice is still lacked in the international community.

Let us look at the example of the United Kingdom in the field of reproduction. How can it establish a regulatory system so that it can even lead the United States in making advances in biological research? The United States may have very complex religious constitutions, making similar development very difficult.

**Sun:** The leadership of the United Kingdom in advancing controversial biological studies is due to its open discussion of cultural aspects of these technologies. I think the crucial point is to base decisions on science and to have sufficient scientific debate on the pros and cons of technologies.

**Duan:** I agree with the importance of having the voice of China in ethical issues. We should strengthen interdisciplinary research on ethical issues. We can set ethical topics as the schedule of scientific research so that researchers can identify ethical issues or ethical conflicts in a particular study and then help

China has already achieved significant progress in science. It has the right of speech in many scientific issues, but its power to express in ethical matters is something we should consider.

—Junjiu Huang

One false impression is such ethics is a burden for research. Benevolently treating animals is necessary to ensure the quality of research data.

—Qiang Sun

form relevant rules and norms to regulate such ethical problems. Through such continuous practices, I think that we can have a voice in the world. Of course, education and communication are also critical.

**Qiu:** The problem is that our ethicists have not sufficiently examined ethical issues in AI practices or other emerging technologies. If we google many ongoing international conferences on AI ethics, how many Chinese ethics researchers can you find? Very few. When you only study histories but do not study these practical issues, how can you say you need to have your voice heard? You do not even know what is going on. You have no right to speak without investigation.

I hope our regulators embrace a global vision. The innovation, R&D and applications of emerging technologies have been unfolding globally. Before drafting the regulations, the debates on ethical issues must be sufficient. So, we should actively participate in international meetings on these topics, and organize more academic seminars on ethical issues in emerging technologies to develop ethics guidelines and legal regulations. Concerning existing international or foreign ethical guidelines, we could draft a tentative standard, and then organize an international conference, inviting external experts to come and discuss together. Slowly, we would formulate our own legally binding regulations or rules with sufficient consideration of the Chinese situation. The question is, we have to do it rather than talking about it. Without actively participating in international debates and agenda-setting, how could this right to speak come automatically? Suppose we issue internationally accessible reports, present our points of view in the international arena, participate in international conferences and publish our articles in internationally prominent magazines. In that case, the international community will gradually hear your voices. The right to speak in science and technology only exists in a participating and collaborative context, not in an isolated setting.

## BUILDING CAPACITY AND IMPROVING PUBLIC AWARENESS FOR RESEARCH ETHICS

**Jia:** Professor Zhai mentioned training. In my personal experience, learning ethical requirements is the first step of taking graduate courses at top international universities. Can China move one step ahead in this direction?

**Zhai:** In implementing new ethical rules, training multiple parties is necessary. The capacity building of the ethics review committees of various agencies is particularly critical. Sometimes this committee may evade their supervision duty, and more often, they are incapable of implementing such function.

We have been stressing that all IRB members should have training before starting their work. In addition to essential content related to ethics review, this training should also involve national security, biosafety and lab safety. Now the critical problem is that these agencies are isolated from each other. MOH, MOST, Ministry of Education and Military departments all have their standards. There should be nationally universal training standards and programs.

But the practices of the training may also be problematic. The consistency between the stance and contents of the textbook is critical. Many organizations that now provide training and certification have limited capacity to train practical skills in ethical governance, and have wasted a tremendous amount of resources to practice the training.

**Jia:** Professor Zhai discussed the training for IRB members. How about ordinary scientists and researchers?

**Qiu:** In addition to IRB members, scientists and regulators who oversee science and technology should be trained.

**Sun:** Currently, ethics training is not done regularly for scientists. The ethics board for the experimental animals in our institute routinely invite ethicists to give lectures, but this is not an obligation.

**Zhai:** Authorities must work out standard training materials. One of the standards should be in line with Chinese laws and regulations, and with Chinese culture. Of course, they should be in line with the basic norms of ethics. Currently, international pressure on China's research ethics often starts from unauthentic reports by journalists. Therefore, ethical training for media staff is also necessary. For the team of pharmaceutical R&D, ethics training is essential too. All of these aspects should be notably improved.

**Sun:** In terms of the ethics for experimental animal use, regulators and scientists should be widely educated to correct two wrong perceptions. One false impression is such ethics is a burden for research. Benevolently treating animals is necessary to ensure the quality of research data. Second, some think we sacrifice animals for the benefits of human beings. This impression is also incorrect. While we use experimental animals to develop drugs and therapies for human diseases, they will help cure animals. Many animals share similar conditions with human beings. In the research process, we also better know the physiological processes in animals. Therefore, the research with animals helps us to treat animals better as well.

**Jia:** Professors Huang and Zeng, as frontier scientists, what kind of suggestions would you have in this regard?

**Huang:** I am in charge of safety in my college now. Putting safety education on the compulsory courses for graduate and undergraduate students is one of our primary goals. But now, safety education focuses more on instrument safety, biosafety, chemical fire prevention and so on. Ethical aspects need to be involved, but so far, little has been done.

**Zeng:** In terms of AI, recently, AI ethics has raised full attention among AI researchers and practitioners. Starting in 2018, Stanford University, MIT and Cornell University opened courses on AI ethics. At the University of Chinese Academy of Sciences (UCAS), we also began to offer AI philosophy and ethics courses to engineering and social science students at UCAS. These courses are essential for AI researchers and practitioners because these students will become AI stakeholders shortly. Starting from 2019, Peking, Zhejiang and Xi’an Jiaotong universities will have relevant courses for master students, and undergraduates majoring in AI.

**Huang:** It is regretful that, according to professor Qiu, some science leaders thought it was not the right time to study research ethics. Hence, we need more people to understand the importance of ethics. The more they know, the more they will support measures on ethical management.

**Sun:** Science literacy in China is not high. I think the practical way is to say that we cannot treat the public as an onlooker. We want the public to know the significance of this matter, and make more people understand it. Hence, it is vital to have science popularization of research ethics.

**Zeng:** In AI, this also involves educating and instructing those whose industries may be strongly impacted by technology. These workers hand over to or share their experience and expertise with robots or machines. We should compensate for them. On the other hand, we should make sure that they are aware of the potential risks and make the necessary preparations.

**Huang:** Science popularization is not just to educate about science ethics at the social level. We should also need to put this content into our higher education. Any person engaged in such cutting-edge research should touch the rules of these values and human subject protection in his/her undergraduate course. If I am already an independent PI, when I am told to study ethics, I may not necessarily listen to it or I may think it has nothing to do with my work.

## Notes

Hepeng Jiais a professor of science communication at Soochow University and a freelancing science writer for NSR
